# Evidence of influenza A infection and risk of transmission between pigs and farmworkers

**DOI:** 10.1111/zph.12948

**Published:** 2022-04-20

**Authors:** Gustavo Lopez‐Moreno, Peter Davies, My Yang, Marie R. Culhane, Cesar A. Corzo, Chong Li, Aaron Rendahl, Montserrat Torremorell

**Affiliations:** ^1^ Veterinary Population Medicine Department, College of Veterinary Medicine University of Minnesota St. Paul Minnesota USA; ^2^ Veterinary and Biomedical Sciences Department, College of Veterinary Medicine University of Minnesota St. Paul Minnesota USA

**Keywords:** farmworkers, influenza, swine, transmission

## Abstract

Interspecies transmission of influenza A virus (IAV) between pigs and people represents a threat to both animal and public health. To better understand the risks of influenza transmission at the human–animal interface, we evaluated 1) the rate of IAV detection in swine farmworkers before and after work during two human influenza seasons, 2) assessed risk factors associated with IAV detection in farmworkers and 3) characterized the genetic sequences of IAV detected in both workers and pigs. Of 58 workers providing nasal passage samples during 8‐week periods during the 2017/18 and 2018/19 influenza seasons, 33 (57%) tested positive by rRT‐PCR at least once. Sixteen (27%) workers tested positive before work and 24 (41%) after work. At the sample level, 58 of 1,785 nasal swabs (3.2%) tested rRT‐PCR positive, of which 20 of 898 (2.2%) were collected prior to work and 38 of 887 (4.3%) after work. Although farmworkers were more likely to test positive at the end of the working day (OR = 1.98, 95% CI 1.14–3.41), there were no influenza‐like illness (ILI) symptoms, or other risk indicators, associated with IAV detection before or after reporting to work. Direct whole‐genome sequencing from samples obtained from worker nasal passages indicated evidence of infection of a worker with pandemic 2009 H1N1 of human‐origin IAV (H1‐pdm 1A 3.3.2) when reporting to work, and exposure of several workers to a swine‐origin IAV (H1‐alpha 1A 1.1) circulating in the pigs on the farm where they were employed. Our study provides evidence of 1) risk of IAV transmission between pigs and people, 2) pandemic H1N1 IAV infected workers reporting to work and 3) workers exposed to swine harbouring swine‐origin IAV in their nasal passages temporarily. Overall, our results emphasize the need to implement surveillance and transmission preventive protocols at the pig/human interface.

## 1 INTRODUCTION

Influenza A virus (IAV) is a multi‐species virus capable of infecting homeothermic vertebrate species including birds, swine and humans. There are documented events of zoonotic IAV transmission from animals to people (Choi et al., 2015; Ma et al., 2019; Rajão et al., 2017) resulting in variable impacts on public health. However, transmission of IAV from people to animals (zooanthroponosis or reverse zoonosis) has also been documented and contributes to IAV genetic diversity in pigs (Nelson et al., 2014). In turn, both human and animal populations may be reservoirs of IAV strains capable of causing infections back to the other species. Ultimately, novel emerging infections that result in new pandemics are the most consequential, as was the case with the most recent influenza pandemic in 2009 (Mena et al., 2016).

Among all the animal–human interfaces, the pig–human interface is of special interest. Pigs and humans can become infected with avian, swine and human‐origin influenza viruses (Nelli et al., 2010; Balzli et al., 2016) because their respiratory tract contains both avian α‐2,3 and mammalian α‐2,6 sialic acid receptors. As a result, pigs are considered a bridge species for the transmission of avian IAVs to humans since they are capable of generating novel viruses with pandemic potential. Pig–human interfaces including agricultural animal fairs, live animal markets and swine farms have documented swine‐to‐human IAV transmission events (Bowman et al., 2014; Choi et al., 2015; Schicker et al., 2016; Ma et al., 2019; Chastagner et al., 2019; Cook et al., 2020). However, the IAV bidirectional transmission events between pigs and people that take place in swine farms are poorly understood. Retrospective studies have shown that farmworkers have higher IAV seroconversion rates than those of people without day‐to‐day contact with pigs (Gray et al., 2007; Terebuh et al., 2010). Moreover, household members of farmworkers, even those who did not have swine contact, also had higher seroprevalence to IAV (Gray et al., 2007). Overall, this information suggests that farmworkers may serve as another important conduit for potential IAV transmission from pigs to communities.

There is also substantial evidence of multiple spill‐over events of human IAVs into swine populations (Rajao et al., 2019; Belser et al., 2011). Nelson et al. documented at least 49 distinct zooanthroponotic introductions of the 2009 pandemic H1N1 viruses into pigs globally in a short period of 2 years. There was also evidence of 23 additional non‐pandemic IAV introductions from human to pigs of H1 and H3 seasonal IAVs (Nelson et al., 2012), and multiple introductions of human‐origin seasonal viruses that have further reassorted with endemic IAVs circulating in swine have been documented in the United States (US) (Nelson et al., 2014). Introductions of human‐origin IAV have further increased the genetic and antigenic diversity of IAV in swine (Rajão et al., 2017), (Krog et al., 2017; Nelson et al., 2015). The on‐going expansion of IAV genetic diversity in pigs represents a challenge to both animal and public health due to the difficulty to control infections with the ever‐changing and diverse influenza A viruses.

Despite the recognition that bidirectional transmission of IAV occurs between people and pigs, little is known about the frequency and method of these transmission events. We lack knowledge regarding swine IAV exposure risk of farmworkers. In particular, we do not yet understand how often workers test positive after being in close contact with pigs nor whether they can become carriers of IAV in their nasal passages. The latter is important to understand given that the aerosol route is considered a main route of IAV transmission (Corzo et al., 2013). Furthermore, we lack knowledge on how frequently farmworkers report to work with IAV infections that may result in spill‐over events to pigs, the IAV genotypes humans carry, and whether there are interventions that can be identified to help reduce the risk of IAV transmission to the pigs.

To advance the understanding of the risk of bidirectional IAV transmission between pigs and people, we studied the pig–human interface in breeding farms, focusing on farmworkers. During the peak period of two human influenza seasons, we evaluated 1) the risk of IAV detection in swine farmworkers prior to and after work, 2) assessed risk factors associated with IAV detection in the workers and 3) characterized the genetic sequences of the IAV detected in both the workers and the pigs. Such fundamental information is essential to further understand the risks of transmission between pigs and people and assess the need to implement mitigation measures at the human–animal interface.

## 2 MATERIALS AND METHODS

### 2.1 Ethics statements

Protocols and procedures were approved by the University of Minnesota Institutional Animal Care and Use Committee (protocol number 1809‐36338A), the Institutional Biosafety Committee (protocol number 1808‐36316H) and the Institutional Review Board (protocol number STUDY00004807). Pigs were owned by producers who had provided written consent to have samples collected from the animals. Farmworkers volunteered to participate in the study, signed informed consent forms and had the option to withdraw from the study at any point in time.

### 2.2 Experimental design

#### 2.2.1 Farm selection

The study was conducted during the 2018 and 2019 winter seasons in the Midwestern United States, a region of diverse agricultural activity including significant swine production. Farmworkers on seven commercial swine breeding herds belonging to five pig production companies located in four Midwestern states volunteered to participate in the study. Breeding herds were selected because they have been shown to be IAV reservoirs (Allerson et al., 2014), typically have more farmworkers than grow‐finish farms, and workers frequently have close interactions with pigs when performing daily tasks that involve pig handling (e.g. assisting farrowing, breeding sows, processing piglets, vaccination or treatments). Herd inventories ranged approximately between 1,500–4,000 breeding females and 2,000–6,000 suckling piglets. All farms had a history of piglets testing IAV positive in the 6 months prior to the start of the study. However, IAV status at the time of enrolment had only been confirmed in 4 of the farms. Farms A, B and C were sampled during the 2017/18 flu season, and farms D, E, F and G were sampled in the 2018/19 flu season.

#### 2.2.2 Farmworker enrolment

Farmworker participation in the study was voluntary, and participants could withdraw from the study at any time. Workers who were 18 years of age or older and who worked for a minimum of 30 h a week were eligible. At enrolment, baseline information was gathered using a questionnaire to obtain demographic information such as name, contact information, gender, age and whether they had received the seasonal influenza vaccine. After enrolment, study participants were assigned an individual identification number to maintain confidentiality known only to the participant and study personnel. Individualized boxes containing 32 sampling kits were sent to each participating farm so that study participants would have the necessary sampling materials. Each sampling kit contained a flocked swab (Copan Diagnostics INC), a vial with universal viral transport (UVT) media (Becton, Dickinson and Company), a questionnaire (one page in length) and a sealable shipment envelope. For kits that were to be used prior to entering the farm, a disposable thermometer (NextTemp®, Medical Indicators) was also provided. Workers received a gift card at the end of the study to compensate them for their willingness to participate. Compensation was proportional to the number of samples collected with a maximum of USD 50 per participant.

#### 2.2.3 Farmworker sample collection

Before study initiation, researchers monitored the Centers for Disease Control and Prevention (CDC) Weekly Influenza Surveillance report tracking influenza activity in the US human population (https://www.cdc.gov/flu). Sampling was initiated once there was a confirmed upward trend in influenza‐like illnesses (ILI) for the four states where the farms were located. Recruitment of study participants took place days to weeks before initiating sampling. Farmworker sampling consisted of two daily self‐collected nasal swabs with the first nasal swab collected at the farm in the morning prior to entering the barns. The second nasal swab was also collected at the farm but at the end of the working day after having completed farm chores but before showering out of the facility. This sampling protocol occurred twice a week, usually Tuesdays and Thursdays, for eight consecutive weeks for a maximum of 32 sampling events or until the participant withdrew from the study. Study personnel provided training to the farmworkers regarding self‐collection of the nasal swabs. Briefly, the procedure consisted of inserting one swab to a depth of two centimetres into both nostrils and rotating it 2 times against the nasal wall. Once collected, the swab was placed in the vial containing UVT media; then, the vial was sealed and placed in the shipping envelope. Workers also recorded their body temperature before entering the farm by placing the disposable thermometer (NexTemp®, Medical Indicators, Hamilton, NJ, USA) under their tongue for 60 seconds and recording the temperature in the questionnaire.

Farmworkers were asked to complete a short questionnaire each time they collected a nasal swab. The questions gathered information on the participant and any household members that may have exhibited ILI (e.g. fever, headache, cough, sneeze and muscular ache). The questionnaires filled out at the end of the day also recorded the work activities and the farm area where the worker spent most of the day.

After sample collection, vials containing the nasal swabs and the questionnaires were placed in a sealed envelope and kept refrigerated at 4°C until they were shipped by courier in insulated containers to the University of Minnesota (UMN) research laboratories. Upon receipt at the UMN research laboratories, the study personnel made aliquots of the samples and stored the aliquots at −80°C until testing.

#### 2.2.4 Pig sample collection

Sampling of pigs was performed in the participating farms at the beginning, middle and termination of the farmworker sampling period. Thirty nasal swabs (95% confidence of detecting at least one positive sample when the prevalence was at least 10%) were obtained from due‐to‐wean pigs (e.g. ~18 days of age) following published procedures (Garrido‐Mantilla et al., 2019).

#### 2.2.5 Sample testing

RNA extraction from samples was conducted using the magnetic bead particle processor procedure (Ambion® MagMAX™AM1836, Viral RNA Isolation Kit; Applied Biosystems), following the product protocols (MagMAXTM‐96 Viral RNA Isolation Kit, n.d.).

With farmworkers samples, RNA from individual samples was first screened using the CDC rRT‐PCR influenza universal test designed to detect all influenza A viruses following published procedures (WHO, 2009). IAV primers were kindly provided by Dr. John Barnes at the CDC. Samples that had a cycle threshold value (ct) less than 40 were considered positive and were further tested using the CDC rRT‐PCR flu panel for subtyping. To ensure that farmworker’s samples contained human DNA and that the sample RNA was not degraded, samples were also tested by rRT‐PCR to detect the human housekeeping RNase P gene (WHO, 2009) (positive control). Samples that had a cycle threshold (ct) >37 were considered not valid and excluded from the analysis.

RNA from swine samples were tested in pools of 3 by farm and collection day using rRT‐PCR that targets the conserved IAV matrix gene using previously described procedures (Slomka et al., 2010; Nirmala et al., 2021). Samples which tested positive by rRT‐PCR (ct <37) were selected for virus isolation using Madin–Darby Canine Kidney (MDCK) cells as described previously (Meguro et al., 1979).

#### 2.2.6 Complete IAVgenome amplification and sequencing

A subset of farmworker (*n* = 30) and swine (*n* = 20) samples, distributed across farms, was selected for IAV whole‐genome sequencing. The subset included worker samples that tested positive using the screening IAV RT‐PCR, including the ones that yielded subtyping results (*n* = 9), and those from study participants with positive consecutive sampling events. The swine samples with the lowest cycle threshold at each sampling point from farms where workers had tested positive were selected from the IAV rRT‐PCR positive samples.

Viral RNA was extracted from positive nasal swabs, used to amplify all eight segments of IAV using a one‐step RT‐PCR as previously described (Nirmala et al., 2021). The purified PCR products were subjected to library preparation using the Illumina DNA Library preparation kit (formerly Nextera Flex DNA Library kit) (Illumina, San Diego, CA) according to the manufacturer’s recommendation. Final pooled libraries were submitted to the University of Minnesota Genomics Center for 75 bp paired‐end cycle sequencing run on the Illumina MiSeq System (Illumina, San Diego, CA). After automated cluster generation, the sequence reads (FASTQ) files were obtained for further analysis using the bioinformatics pipeline developed at the University of Minnesota Veterinary Diagnostic Laboratory including trimming, denovo assembly, annotation and generation of FASTA sequences of assembled genomes (Nirmala et al., 2021).

#### 2.2.7 Sequence analysis

Clade classification for each segment was done using the automated classification tool OctoFLU (Chang et al., 2019) (Zeller et al., 2021). The raw assembled sequences were manually sorted into eight FASTA files based on their annotation to the IAV segment. FASTA files were then aligned with deposited IAV sequences from the Influenza Research Database (IRD) (Squires et al., 2012) and reference sequences from OctoFLU using the MUSCLE alignment tool within Geneious (2021.0.3) (https://www.geneious.com) (Geneious | Bioinformatics Software for Sequence Data Analysis). Phylogenetic trees were built for each IAV segment using a maximum likelihood method in IQtree program (IQ‐TREE: Efficient Phylogenomic Software by Maximum Likelihood), with the best‐fitting nucleotide substitution model, in 1000 bootstrap replicates. Trees were assembled via the Minnesota Supercomputing Institute (MSI) platform and edited using the interactive tree of life (iTOL) online tool (Letunic & Bork, 2019).

#### 2.2.8 Data analysis

Data collected from the questionnaires and the quantitative rRT‐PCR results were consolidated in a spreadsheet (Microsoft Excel, Microsoft Corporation) for analysis. To identify potential risk factors associated with farmworker IAV status, the association of the different variables with the rRT‐PCR results was investigated using univariate generalized linear models using R statistical software (version 4.1.1) (R Core Team, 2017) (Appendix S13). Then, the variables that had a p‐value <0.20, or that were of special interest or that were relevant to the aims of the study, were selected and included in a logistic mixed‐effects model using the glmer function in R statistical software (version 4.1.1) (R Core Team, 2017) to evaluate the association between the variables and the probability of workers testing IAV‐RNA positive. Time of sample collection, having ILI, vaccination status and farm area where the work was performed were variables included as fixed effects. Farm and participant identification were added as random effects to account for the lack of independence between samples obtained from the same farm and participants.

## 3 RESULTS

### 3.1 Demographic and information of farmworkers

**FIGURE 1:**
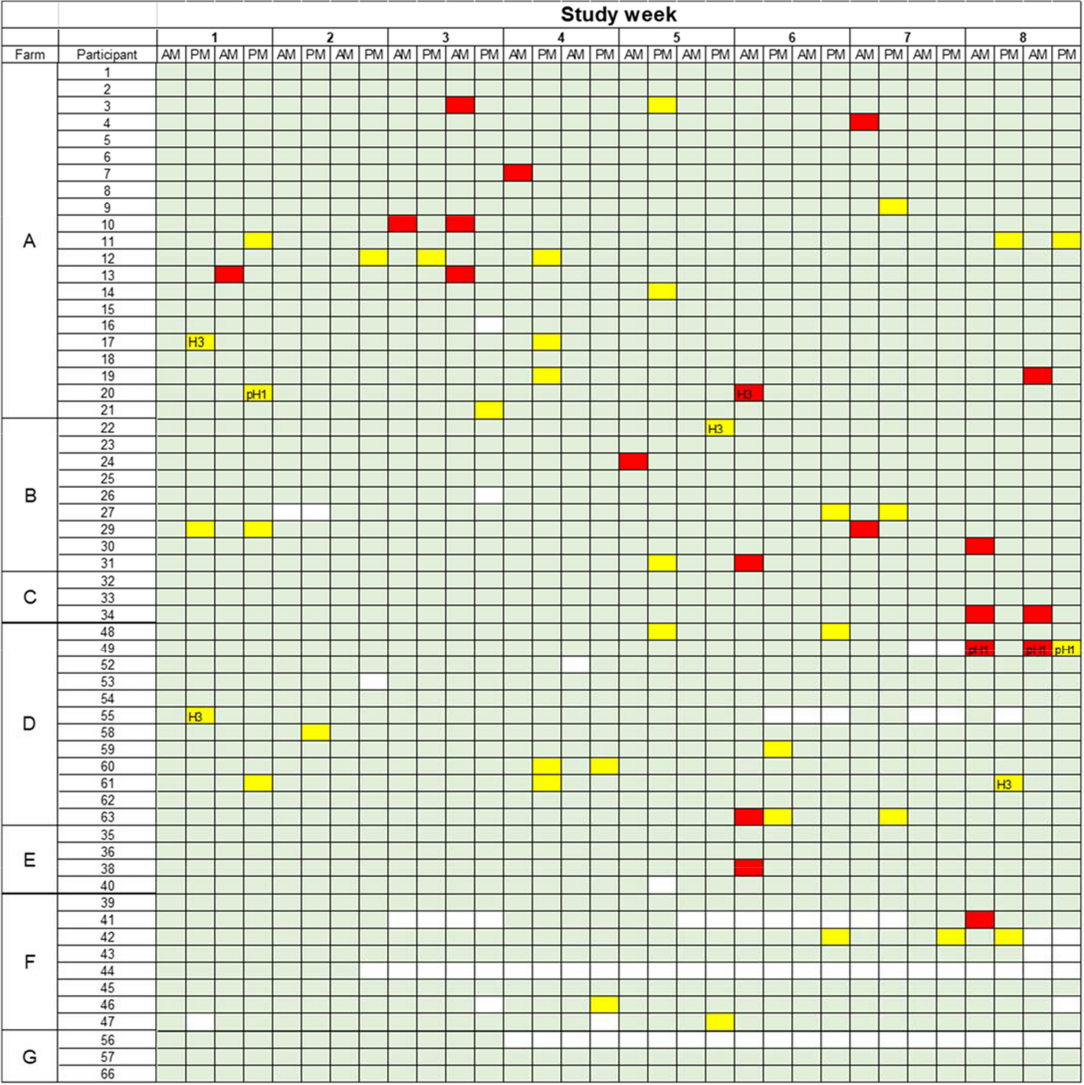
Distribution of individual influenza a virus (IAV) rRT‐PCR results from participants by farm and time of collection. AM: Samples were collected prior to entering the workplace; PM: Samples were collected after work. Light green squares depict collected samples that tested IAV negative. Red squares represent IAV‐RNA positive samples collected before entering the swine farm. Yellow squares represent IAV‐RNA positive samples collected after working in the farm. Blank squares represent samples not collected due to participant dropout (participants 44 and 56) or participant not reporting to work on that day. H1 and H3 results, if obtained from the subtyping rRT‐PCR, are shown within the yellow or red squares

Sixty‐four participants from 7 different swine farms enrolled in the study, and their baseline information is summarized in Table 1. Four participants withdrew from the study after signing the consent form and did not contribute any samples. Two additional participants withdrew from the study before completing less than half of the samplings. Forty‐five (70%) participants completed the sampling protocol, whereas thirteen (20%) had some missing samples (Figure 1).

**TABLE 1:** Number (%) of enrolled participants by farm, gender and seasonal influenza vaccination status in seven breeding herds in midwestern United States

Farm	Number of participants	Gender (%)		Influenza vaccinated (%)	Age range
		Male	Female	Yes	Min–max
A	21	16 (76)	5 (24)	15 (71)	21–60
B	10	7 (70)	3 (30)	3 (30)	25–43
C	3	3 (100)	0 (0)	2 (67)	25–34
D	12	6 (50)	6 (50)	1 (8)	21–44
E	4	3 (75)	1 (25)	0 (0)	38–59
F	8	6 (75)	2 (25)	1 (13)	21–53
G	6	1 (17)	5 (83)	2 (33)	22–50
Total	64	42 (66)	22 (34)	24 (38)	21–60

### 3.2 Swine and farmworker rRT‐PCR IAVresults

**FIGURE 2:**
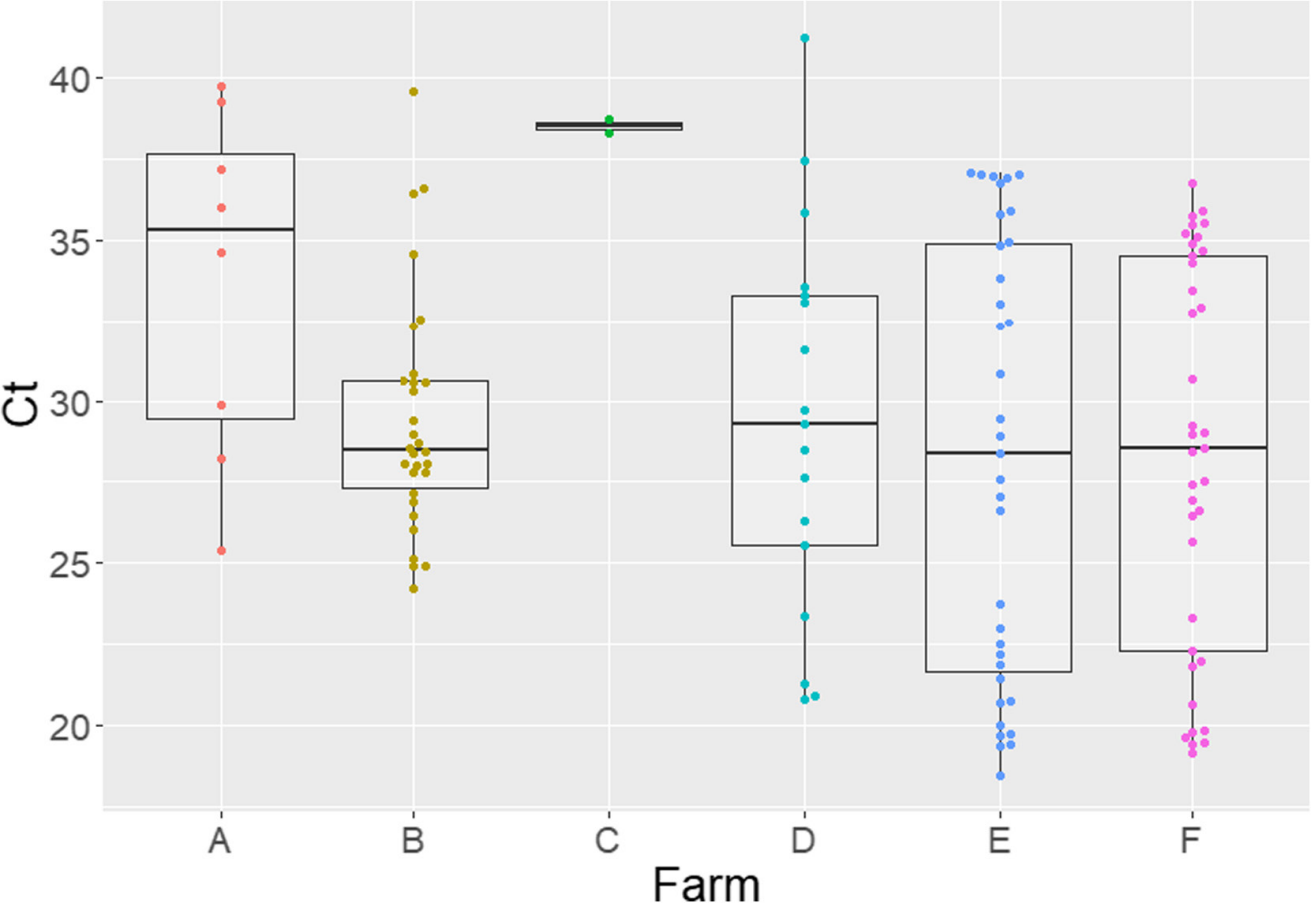
Distribution of influenza a virus rRT‐PCR cycle threshold (ct) values obtained from swine nasal swabs by farm (*x*‐axis). Ct values (*y*‐axis) lower than 37 were considered positive

Only five farms had rRT‐PCR positive piglets during the study (Figure 2). Farms B, D, E and F had piglets testing IAV positive at weaning in all 3 sampling points during the course of the study. Farm A had piglets testing IAV positive only once at the middle point of the farmworkers sampling. Piglets from farms C and G did not test IAV positive at any sampling point.

**FIGURE 3:**
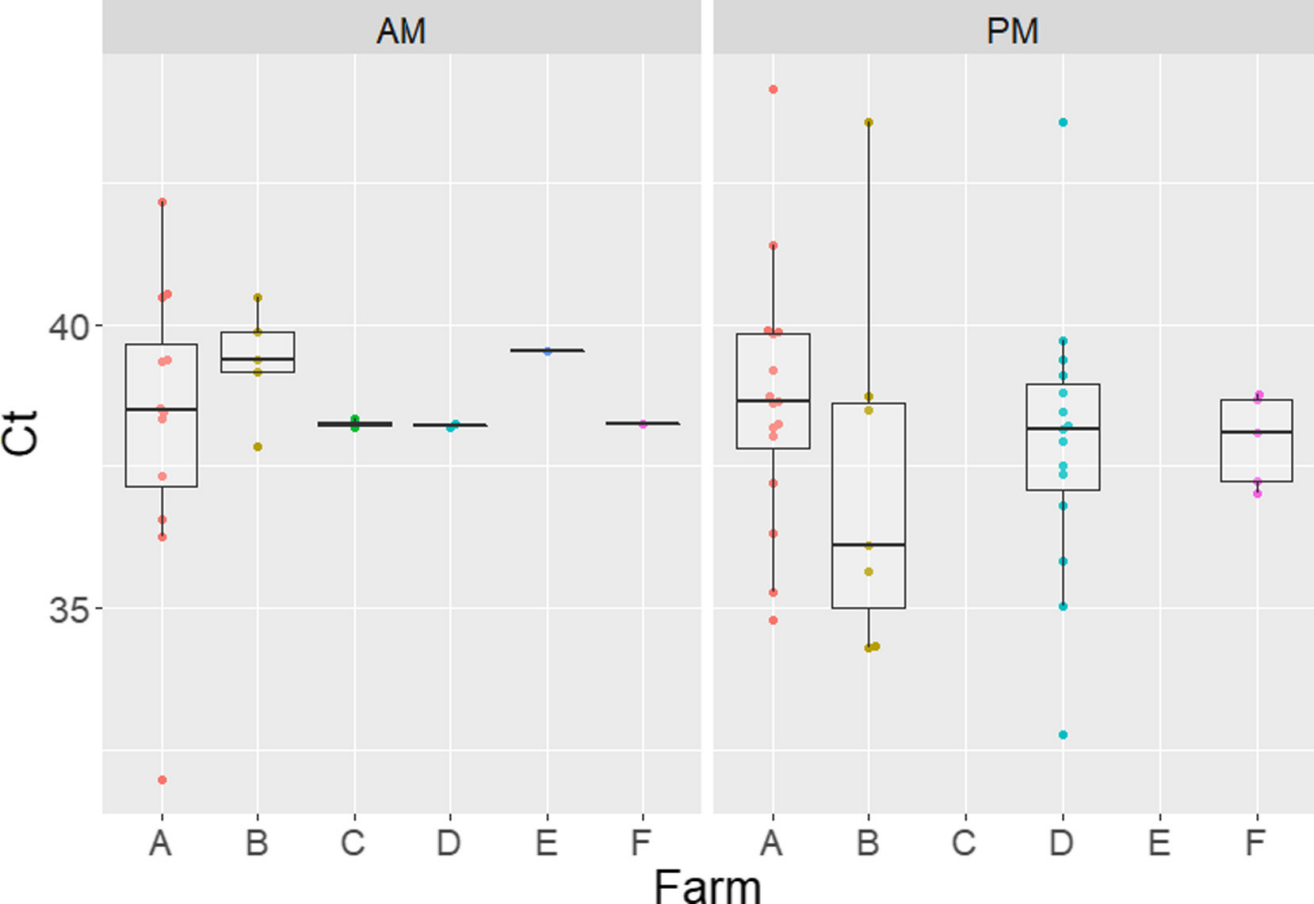
Distribution of influenza a virus cycle threshold (Ct) rRT‐PCR values obtained from farmworkers by time of sample collection and farm. Ct values lower than 40 were considered positive. There was no IAV detection in farmworkers from farm G. AM represents the samples collected before work and PM represents the samples collected after work

A total of 1,814 nasal swabs were collected from participating farmworkers. The human RNase P housekeeping gene was detected in 98.4% of the samples with ct values ranging from 22 to 42 (Appendix S1). Participant samples that tested negative for the RNase P gene (*n* = 29) were removed from the study. From the remaining 1,785 nasal swabs, 58 (3.2%) tested positive for IAV rRT‐PCR (Table 2) and 33 (56.9%) workers tested positive on at least one occasion (Table 3) with ct values ranging between 31.9 and 39.8 (Figure 3). Six of the seven (86%) farms had at least one worker who tested IAV‐RNA positive during the course of the study. There were no differences in detection rates (*p* = .51) between the 2017/18 and the 2018/19 seasons (data not shown).

**TABLE 2:** Number (%) of influenza a virus (IAV) and RNase P gene rRT‐PCR positive nasal swabs from workers in seven breeding farms in the midwestern United States

Farm^a^	Number of qualifying swabs^b^	Number of IAV‐RNA positive nasal swabs (%)	Number of IAV‐RNA positive nasal swabs before farm entry (%)	Number of IAV‐RNA positive nasal swabs after farm work (%)	Number of RNase P positive nasal swabs^c^ (%)
A	665	23 (3.5)	9/336 (2.7)	14/329 (4.3)	665/671 (99.1)
B	279	10 (3.6)	4/140 (2.9)	6/139 (4.3)	279/285 (97.9)
C	70	2 (2.9)	2/35 (5.7)	0/35 (0)	70/71 (98.6)
D	360	16 (4.4)	3/180 (1.7)	13/180 (3.6)	360/374 (96.3)
E	127	1 (0.8)	1/64 (1.6)	0/63 (0)	127/127 (100)
F	208	6 (2.9)	1/105 (0.9)	5/103 (4.9)	208/210 (99)
G	76	0 (0)	0/38 (0)	0/38 (0)	76/76 (100)
Total	1,785	58 (3.2)	20/898 (2.2)	38/887 (4.3)	1,785/1,814 (98.4)

^a^ Farms A, B and C were sampled during the 2017/18 influenza season and farms D, E, F and G during the 2018/19 influenza season.

^b^ Total number of swabs collected that tested RNase P positive.

^c^ Number of nasal swabs positive by rRT‐PCR/total samples collected.

**TABLE 3:** Number (%) of influenza a virus (IAV) rRT‐PCR positive farmworkers by farm

Farm	Number of workers	Number of workers IAV‐RNA positive^a^ (%)	Number of workers IAV‐RNA positive before farm entry (%)	Number of workers IAV‐RNA positive after farm work (%)
A	21	13 (61.9)	7 (33.3)	9 (42.9)
B	9	6 (66.6)	4 (44.4)	4 (44.4)
C	3	1 (33.3)	1 (33.3)	0 (0)
D	12	8 (66.7)	2 (16.7)	8 (66.7)
E	4	1 (25)	1 (25)	0 (0)
F	7	4 (57.2)	1 (14.3)	3 (42.8)
G	2	0 (0)	0 (0)	0 (0)
Total	58	33 (56.9)	16 (27.3)	24 (41.4)

^a^ Number of participants that tested IAV‐RNA positive at least once during the course of the study.

Of the samples collected prior to work (i.e. AM; *n* = 898), 20 (2.2%) samples from 16 (27.3%) workers tested rRT‐PCR positive. Of these 16 workers, 12 tested IAV‐RNA positive only once whereas four tested positive twice. Of the samples collected after work (i.e. PM; *n* = 887), 38 (4.24%), those belonging to 24 (41.4%) workers tested IAV‐RNA positive. Of these 24 workers, 14 tested positive once and 10 tested positive at least twice. On six occasions, workers with multiple positive results tested positive twice within a given week (Figure 1).

Nine of the rRT‐PCR positive samples that were tested with the subtyping PCR panel provided by the CDC were positive. Five samples from five distinct workers were positive to the H3 subtype (1 from before work and 4 after work), while four samples from two distinct workers were positive to the 2009 pandemic H1 subtype. One was confirmed positive by whole‐genome sequencing (see below), while the other tested positive once at the end of the working day. No samples yielded virus via the virus isolation assay.

### 3.3 Risk factors analysis and IAVin the workers

Table 4 summarizes the ILI reported by workers reporting to work and their IAV rRT‐PCR results at the corresponding times. Of the 898 samples collected prior to work, 285 (31.7%) samples originated from farmworkers reporting at least one ILI symptom such as coughing, sneezing, muscular ache, sore throat and/or fever. Of the 20 IAV‐RNA positive samples collected before entering the swine farm, three (15%) belonged to one farmworker who reported at least one ILI symptom, and from whom a 2009 pandemic‐like H1N1 (1A.3.3.2) was detected. Furthermore, eight (40%) of the twenty positive samples collected prior to farm entry originated from workers who had received the seasonal influenza vaccine. Body temperature obtained using the disposable thermometer showed nine sampling events in which workers reported oral temperatures above the fever threshold of 100.4°F. However, none of the corresponding samples tested IAV‐RNA positive by rRT‐PCR.

**TABLE 4:** Summary of influenza‐like illness (ILI) symptoms reported by workers in seven breeding herds prior to entering the workplace by influenza a virus (IAV) rRT‐PCR results of the nasal swab samples collected at the time of survey completion

Question	Number answered yes (%)	Number positive with symptom (%)	Number positive without symptom (%)	*p*‐value^a^
Fever	11 (1.2)	0/11 (0)	20/887 (2.3)	.99
Cough	195 (21.7)	3/195 (1.5)	17/703 (2.4)	.59
Sneeze	131 (14.6)	0/131 (0)	20/767 (2.6)	.09
Muscular ache	97 (10.8)	0/97 (0)	20/801 (2.5)	.15
Household ILI symptoms	99 (11)	1/99 (1)	19/799 (2.4)	.72
Thermometer‐based fever^b^	6 (0.7)	0/6 (0)	20/892 (2.2)	.99

^a^ *p*‐value obtained using Fisher exact test for proportions between the number of positives with and without symptoms.

^b^ Results obtained based on the disposable thermometer used at the time of collecting the samples prior to enter the farm. A thermometer reading ≥100.4°F was considered indicative of fever.

From the 887 samples collected at the end of the working day, 255 (28.7%) were obtained from farmworkers reporting at least one ILI symptom (Table 5) and of the 38 IAV‐RNA positive samples detected after working in the farm, only 7 (18.4%) belonged to farmworkers reporting at least one ILI symptom.

**TABLE 5:** Summary of influenza‐like illness (ILI) symptoms reported in the questionnaires by the workers after working in the swine farm distributed by influenza a virus (IAV) rRT‐PCR results of the nasal swabs collected at the time of completing the exit survey

Question	Number answered yes (%)	Number positive with symptom (%)	Number positive without symptom (%)	*p*‐value^a^
Fever	7 (0.8)	0/7 (0)	38/880 (4.3)	.99
Cough	194 (21.9)	3/194 (1.5)	35/693 (5.1)	.04
Sneeze	129 (17)	2/129 (1.5)	36/758 (4.7)	.15
Muscular ache	112 (12.6)	6/112 (5.4)	32/775 (4.1)	.61

^a^ *p*‐values obtained using Fisher exact test for proportions between the number of positives with and without symptoms.

Results of the mixed generalized linear model (Table 6) indicated that after work, farmworkers were twice as likely to test IAV‐RNA positive (OR = 1.98, *p* = .01) after adjusting for ILI symptoms, farm area, IAV vaccination status and farm IAV status and that farm area where the workers had worked during the day did not matter (*p* = .2).

**TABLE 6:** Odds ratios (OR) of farmworkers testing influenza a virus (IAV) positive using a logistic mixed‐effects model

Variable	Category	Number of positive samples/total samples (%)	Β^a^	SE^b^	OR (95% CI)^c^	*p*‐value
Time of collection	Before work	20/898 (2.2)	Reference		–	–
	After work	38/887 (4.3)	0.68	0.28	1.98 (1.15–3.42)	.01
ILI Symptoms	No	48/1,249 (3.8)	Reference		–	–
	Yes	10/536 (1.9)	−0.73	0.37	0.45 (0.23–0.99)	.03
Farm area	Breeding	14/360 (3.9)	Reference		–	–
	Farrowing	26/717 (3.6)	−0.05	0.37	0.95 (0.46–1.96)	.89
	Mix	18/708 (2.5)	−0.35	0.39	0.70 (0.33–1.51)	.37
IAV vaccinated	No	36/1071 (3.4)	Reference		–	–
	Yes	22/714 (3.1)	0.01	0.29	1.01 (0.57–1.78)	.96
Farm IAV status	Negative	2/132 (1.5)	Reference		–	–
	Positive	56/1,653 (3.4)	0.94	0.75	2.55 (0.58–11.13)	.2

Note Time of collection, influenza‐like illness (ILI) symptoms, farm area, vaccination status and farm IAV status were added as fixed effects. Farm and participant identification were added as random effects.

^a^ Model estimate.

^b^ Standard error.

^c^ Odds ratio (95% confidence interval).

### 3.4 Whole‐genome sequencing

**FIGURE 4:**
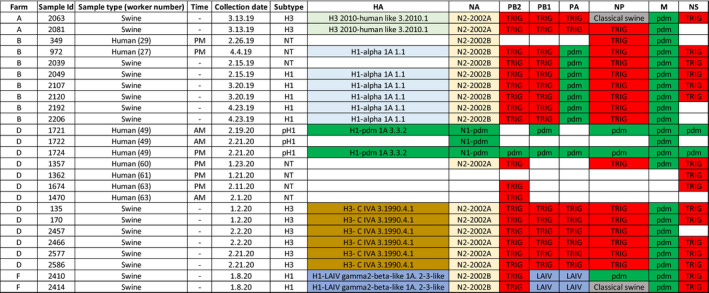
Clade classifications of the influenza a virus genetic segments from human and swine samples using the OctoFLU platform. Each row is a sample. AM: Samples were collected prior to entering the workplace; PM: Samples were collected after work. pH 1: Samples subtyped as pandemic‐like H1; NT: non‐typeable; HA: hemagglutinin; NA: neuraminidase; PB2: polymerase basic 2; PB1: polymerase basic 1; PA: polymerase acid; NP: nucleoprotein; M: matrix; NS: non‐structural. Gene segments were classified in different clades and coloured as follows: Pandemic‐like clade (green), H1‐alpha clade 1A.1.1 (light blue), H3‐2010‐human‐like clade 1 march 2010 (light green), H3‐cluster IV clade 4 march 1990.1 (light brown), live attenuated influenza vaccine clade 1A.2‐3‐like (blue), N2‐2002A and N2‐2002B (light yellow), triple reassortant internal gene (TRIG; red) and classical swine (grey). Blank squares are segments from which no sequences were obtained. Number in parenthesis in the sample type column refers to the worker identification

Of the 30 human samples submitted for whole‐genome sequencing, partial IAV genome sequences were obtained from 9 samples and whole‐genome assembly was accomplished in only two of the samples (Figure 4). In one of the samples (sample #1724), all gene segments were identified as 2019/2020 human seasonal H1N1, that is a 2009 pandemic‐like H1N1, within clade 1A.3.3.2 (Appendices S2–S12). The other sample (Sample #972) had all gene segments of the H1N1 virus detected belonging to the alpha 1A. 1.1 swine‐origin clade that had also been identified during the study in pigs from the farm, wherein the worker was employed (Appendices S2–S11). Samples from five farmworkers (samples 349, 1,357, 1,362, 1,674 and 1,470), which had only some segments sequenced, had IAV detected whose clade classifications were identical to the clade classifications of the IAV detected in the pigs from the farms in which they worked (Appendices S2–S11).

Twelve complete genome and four partial genome sequences were obtained from the swine samples. From the swine samples, the following five HA clades were identified from four distinct farms in the study: H1‐pdm 1A 3.3.2, H1‐alpha 1A 1.1, H1 LAIV gamma2‐beta‐like 1A 2.3‐like, H3 2010‐human‐like 1 March 2010 and H3 C‐IVA 4 March 1990.1 (Figure 4).

## 4 DISCUSSION

Transmission of IAV between pigs and people represents a threat to both animal and public health. Although bidirectional transmission of IAV between pigs and farmworkers has been documented, there is limited information on how often these events occur and what factors contribute to such transmission. As a first step to investigate the risk of IAV transmission between pigs and people, we evaluated 1) how often workers on pig farms have detectable IAV genetic material in their nasal passages, 2) what type of IAV strains can be detected in the workers, and 3) whether there are risk factors that can help us identify IAV positive workers. We found evidence of a) asymptomatic farmworkers testing IAV‐RNA positive in samples collected both before and after the workday, b) swine‐origin IAV strains present in the nasal passages of workers after work, and c) at least one worker reporting to work infected with human seasonal‐H1N1 IAV of pandemic origin. However, we were unable to identify any specific risk factor or ILI symptom associated with IAV detection, likely due to the low incidence of IAV‐RNA detection in the workers.

It is noteworthy that in six of the seven participating farms, at least one worker tested IAV‐RNA positive when reporting to the workplace and the proportion of workers testing IAV‐RNA positive among farms ranged between 14.3% and 44.4% over the 8 week testing periods. Although the overall detection of IAV‐RNA positive samples at farm entry may seem low at 2% (95% CI 1.2–3.2), when taking into account the amount of farmworkers that report to work daily, with a conservative approach of using the lowest value of the 95% CI, we calculate that over a 90 day period of time approximating a human‐flu season, the likelihood that least 1 farmworker testing IAV‐RNA positive at farm entry is a certainty.

In most of the cases where workers tested positive at farm entry, IAV detection was sporadic. Only one worker tested positive in an IAV negative farm, three workers tested positive in consecutive samplings and only one worker had a human seasonal H1N1 virus, confirmed to be 2019/2020 human seasonal H1N1 belonging to 2009 pandemic‐like clade 1A.3.3.2 as determined by full genome sequencing of the IAV identified in the farmworker’s nasal passages. Although we could not isolate virus from any of the worker samples, participant number 49, from whom the IAV complete genome was sequenced, had mild ILI symptoms that did not, however, prevent the worker from reporting to work and performing chores. Participant 49 tested IAV‐RNA positive in three consecutive sampling events using both the screening and the subtyping RT‐PCR and all gene segments of the worker’s IAV‐RNA positive samples matched the 2019/2020 human seasonal H1N1 based on the Genebank database BLAST results. Furthermore, the IAV circulating in the pigs where participant 49 worked had an H3N2 IAV subtype detected from the pig samples. This is strong evidence that this worker was infected with a human seasonal H1N1 IAV when reporting to work and did not acquire IAV from exposure to pigs on the farm. Transmission of 2009 pandemic‐like H1N1 IAV from people to pigs has been documented on multiple occasions (Nelson et al., 2012; Nelson & Vincent, 2015) or suspected in specific case report investigations (Schaefer et al., 2015). However, presented herein is the first reported detection, to our knowledge, of the full genome sequences of IAV detected in farmworkers as they arrived at the farm in the morning prior to commencing work.

For the majority of the workers, detection of IAV could not be related to infection or to specific ILI symptoms. This lack of relationship makes the IAV‐RNA positive results difficult to interpret. It is important to note that of the 20 samples that tested IAV‐RNA positive at farm entry, none had a positive result on the preceding sampling event conducted after work, which would have occurred 2–5 days earlier. However, because sampling was not done daily, we cannot rule out the possibility that exposures to IAV from pigs occurred the previous day at the farms, and that the detections at the farm entry could be attributable to residual genetic material from those exposures. In addition, because asymptomatic carriage of influenza occurs (Cohen et al., 2021), it is important to consider that workers with PCR positive results that lack symptoms could still be a risk for introducing new strains that could infect pigs, although in our study, the sequencing results from swine samples did not reveal evidence that human‐to‐swine infections had occurred. Whether it was due to the sampling protocol, lack of daily samples, presence of immunity in the participants, limited IAV replication in the nostrils, lack of complete genome sequences obtained or sensitivity of the rRT‐PCR technique, we could not determine conclusively the origin of the genetic material identified in workers reporting to work in most of the cases. Only in one case, did we confidently conclude that the worker reported to work infected with IAV during the 8‐week observation period of the flu season.

There was a higher prevalence of IAV‐RNA positivity in the samples from workers collected at the end of the day, after having worked with pigs, than in samples collected before work. However, the likelihood for samples testing IAV‐RNA positive after work remained low. This is supported by the fact that there were no IAV‐RNA positives in workers after work from the two farms where pigs tested IAV negative. Additional support was obtained by matching a full genome sequence obtained from the nasal passage of a worker (participant 27) to the IAV circulating in pigs in farm B. There was further evidence of IAV‐RNA detected in pigs matching the IAV gene segments in other workers (participants 29, 60, 61 and 63), but in those cases, only partial sequences were obtained. There were only two workers who had positive samples after work who also tested positive at farm entry on the same day. For one of the workers (participant 49), we had evidence that he was infected with a human seasonal H1N1 IAV, but for the other worker (participant 63), we were unable to obtain a sequence that would have helped determine the IAV source. Overall, our results show evidence of deposition in the nares of workers indicating exposure, likely through the aerosol route, of swine‐origin IAV in farm employees during the workday. Isolation of infectious IAV in the air of swine farms has been documented (Neira et al., 2016), and our results highlight that farmworkers can be continually exposed to swine‐origin IAV that circulates in farms, reinforcing the importance of using personal protective equipment while working in the farm.

Virus isolation was not successful on the nasal swabs from workers most likely due to the presence of non‐viable viral content. It is also possible that immunity of farmworkers by being previously exposed to the farm circulating IAV, limited viral replication, and therefore, no viable virus was detected in our samples. We note that our sample collection and transport protocols were not optimized for virus viability preservation, which is necessary to fully assess the risk of transmission between animals and people.

Our results suggested lack of specificity of screening methods based on self‐reporting symptoms, including monitoring body temperatures using disposable thermometers. For the majority of responses, only a few had an IAV‐RNA positive sample associated with the presence of at least one ILI symptom. On the contrary, several samples tested positive when responses indicated absence of ILI symptoms. Contrary to what was expected, the statistical analysis showed a lower odds ratio for testing IAV‐RNA positive in those workers having at least one ILI symptom. Moreover, we found no significant association between the history of influenza vaccination and the rRT‐PCR results obtained from farmworkers. Pre‐existing immunity to IAV within farmworkers at the time of the study was not known, and it was outside the scope of the study to evaluate the impact of antibody levels on IAV detection and whether exposure resulted in an increase in antibody response, which has been reported in previous studies (Gray et al., 2007). Nevertheless, vaccination against IAV in farmworkers should still be encouraged to minimize the risk of becoming clinically ill with IAV of either human or swine origin (What Are the Benefits of Flu Vaccination? | CDC, n.d.).

A point to consider is that this study was conducted during the peak of the human influenza seasons; therefore, our results may overstate the risk of workers being a source of infection to the pigs over the course of a year. However, we do not think that this is the case given the difficulty in detecting and confirming IAV infections in the workers. We believe our results provide conservative reasonable estimates of how often IAV infected workers report to work. It is likely that season‐to‐season differences exist and risk of new strain introductions will depend on the IAV prevalence in the communities, the seasonal vaccination rates in the workers and the matching of the seasonal human influenza vaccines against the circulating strains. On the contrary, our results may overestimate the level of exposure reported in the workers because we purposely selected farms with circulation history of IAV in the pigs. Thus, results from this study need to be interpreted carefully because they may not apply to farms with different prevalence of variants of IAV in the pigs or with different pig populations (i.e. growing/fattening farms or boar studs), or farms that vaccinate to control influenza in the pigs. The main question that still needs to be answered is, ‘How many of the confirmed exposure events result in transmission and infections between pigs and people?’. Interspecies transmission is ultimately, the event that needs to be prevented from occurring if both human health and pig well‐being are to be improved. However, this is a daunting task and outside the scope of our study. Nevertheless, we were able to establish an IAV surveillance system at the human–swine interface that could help further investigate events of potential bidirectional transmission of IAV.

In summary, results from this study should serve as a motivation and the tools herein can be used to design and implement mitigation measures that prevent the bidirectional transmission of IAV between pigs and people. Although the likelihood for a given worker to test IAV‐RNA positive in a given day was relatively low, the overall risk to/from workers cannot be underestimated because of the work schedule and activities of the workers taking place almost on a daily basis. We showed evidence that workers can be asymptomatic carriers of swine‐origin IAV in their nasal passages and confirmed that workers can report to work infected with IAV of human origin that could potentially be transmitted to the pigs. Overall, our study provides evidence of exposure and risk of IAV transmission between pigs and people and emphasize the need to have biosecurity measures in place to prevent IAV transmission between pigs and people. Preventing transmission at the pig/people interface will not only benefit public health and prevent zoonotic infections of pandemic potential, but will also help the swine industry to mitigate the economic impact of IAV infections in pigs.

## CONFLICT OF INTEREST

The authors declare no conflict of interest.

## Supporting information


Appendix S1‐S13

## Data Availability

The data that support the findings of this study are openly available if requested.
