# Supplementary material for: Evidence of influenza A infection and risk of transmission between pigs and farmworkers
Source: Zoonoses Public Health. 2022 Apr 20;69(5):560–71. doi: 10.1111/zph.12948 (PMC9546022; doi:10.1111/zph.12948)
Supplement: Supplementary file 1 — Appendix S1‐S13 [file ZPH-69-560-s001.docx]

Appendix S1.Distribution of RNase P gene cycle threshold (Ct) values obtained from farmworkers by rRT-PCR.


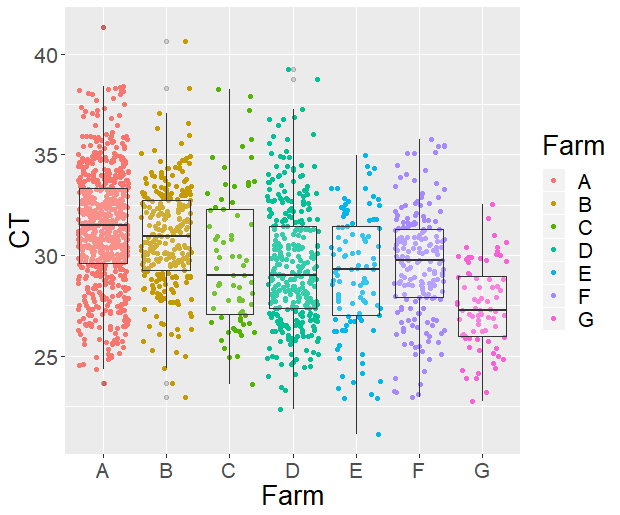


Appendix S2. Hemagglutinin 1 gene (HA1) phylogenetic tree of study samples and references strains.


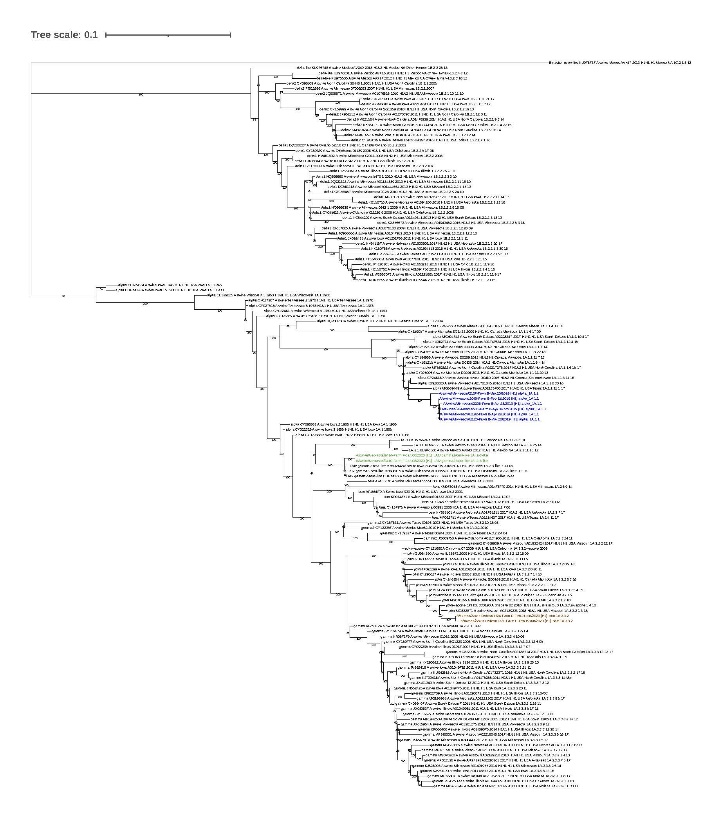


Appendix S3. Hemagglutinin 3 gene (HA3) phylogenetic tree of study samples and references strains.


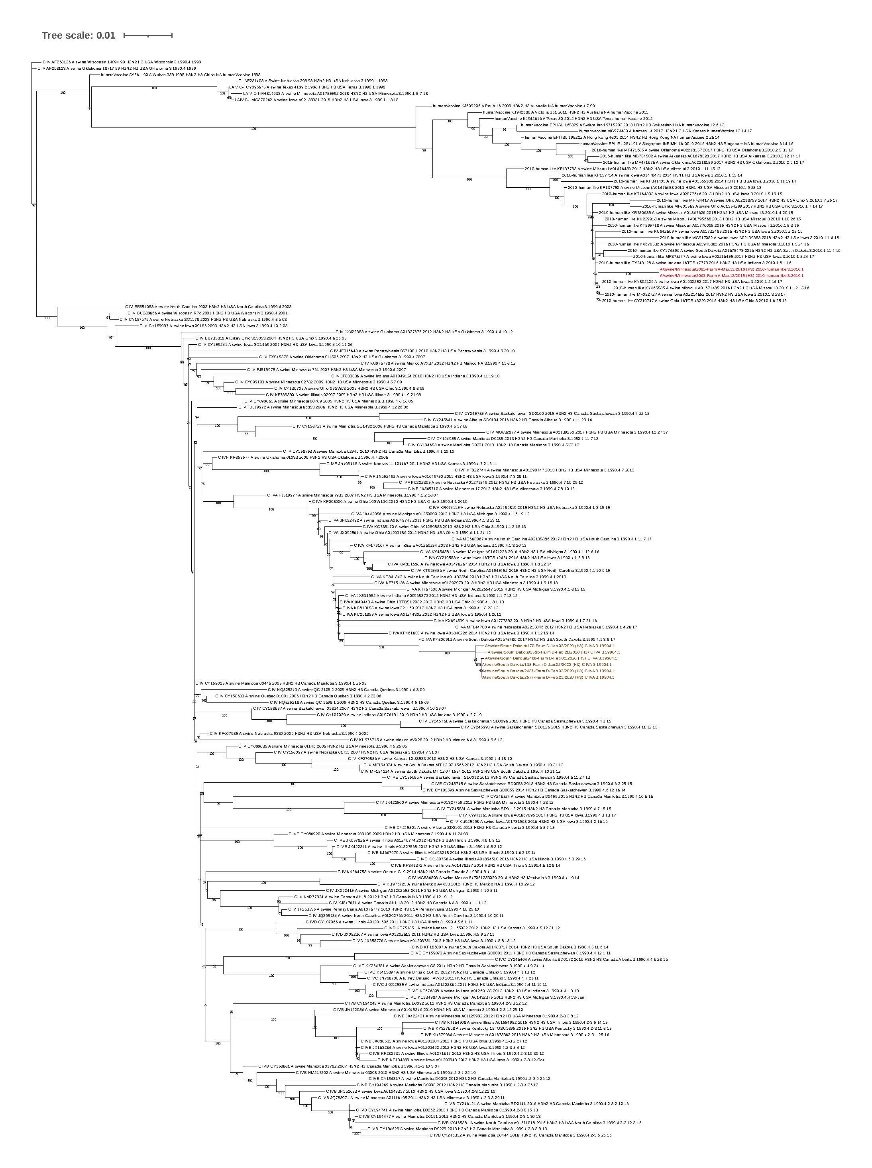


Appendix S4. Matrix gene (M) phylogenetic tree of study samples and references strains.


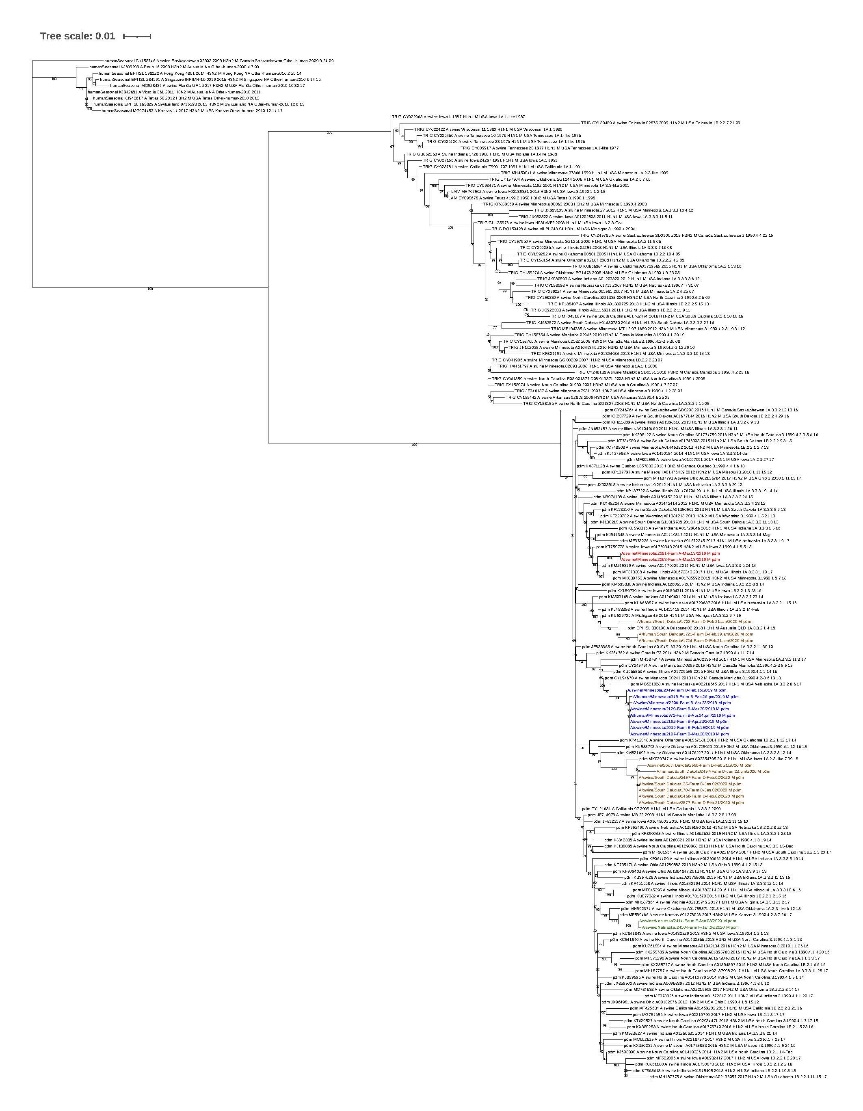


Appendix S5. Neuraminidase 1 gene (NA1) phylogenetic tree of study samples and references strains.


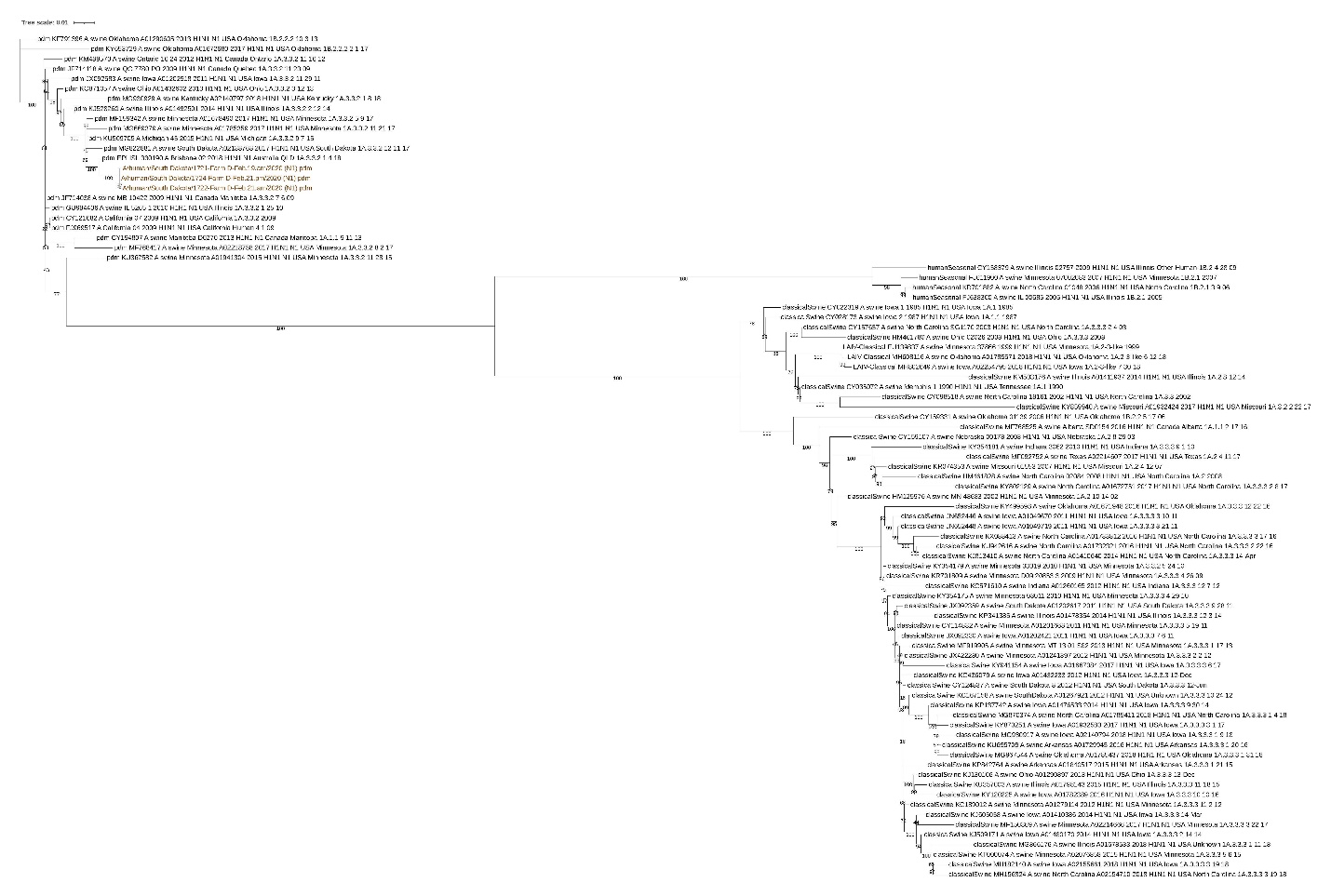


Appendix S6. Neuraminidase 2 gene (NA2) phylogenetic tree of study samples and references strains.


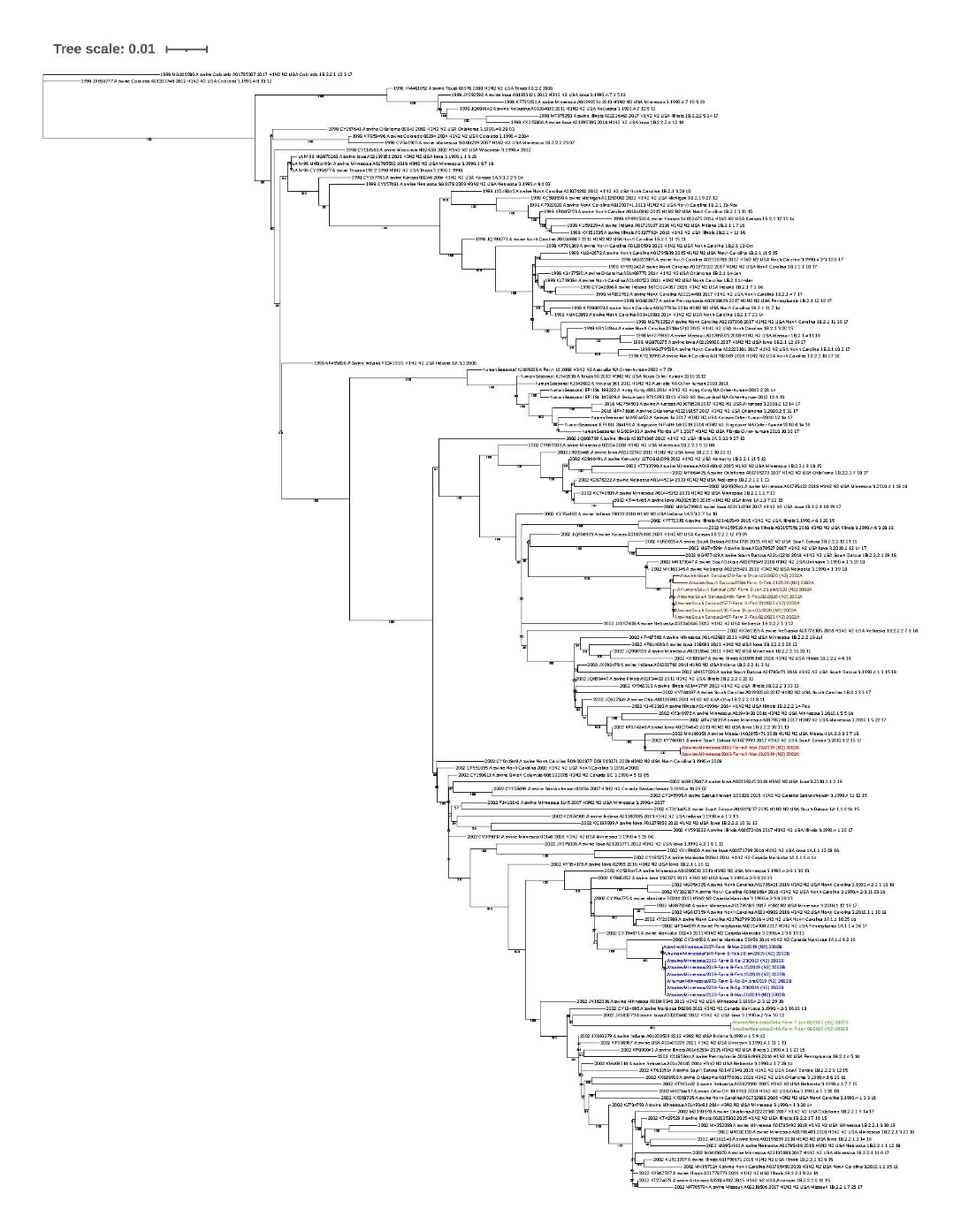


Appendix S7. Nucleoprotein gene (NP) phylogenetic tree of study samples and references strains.


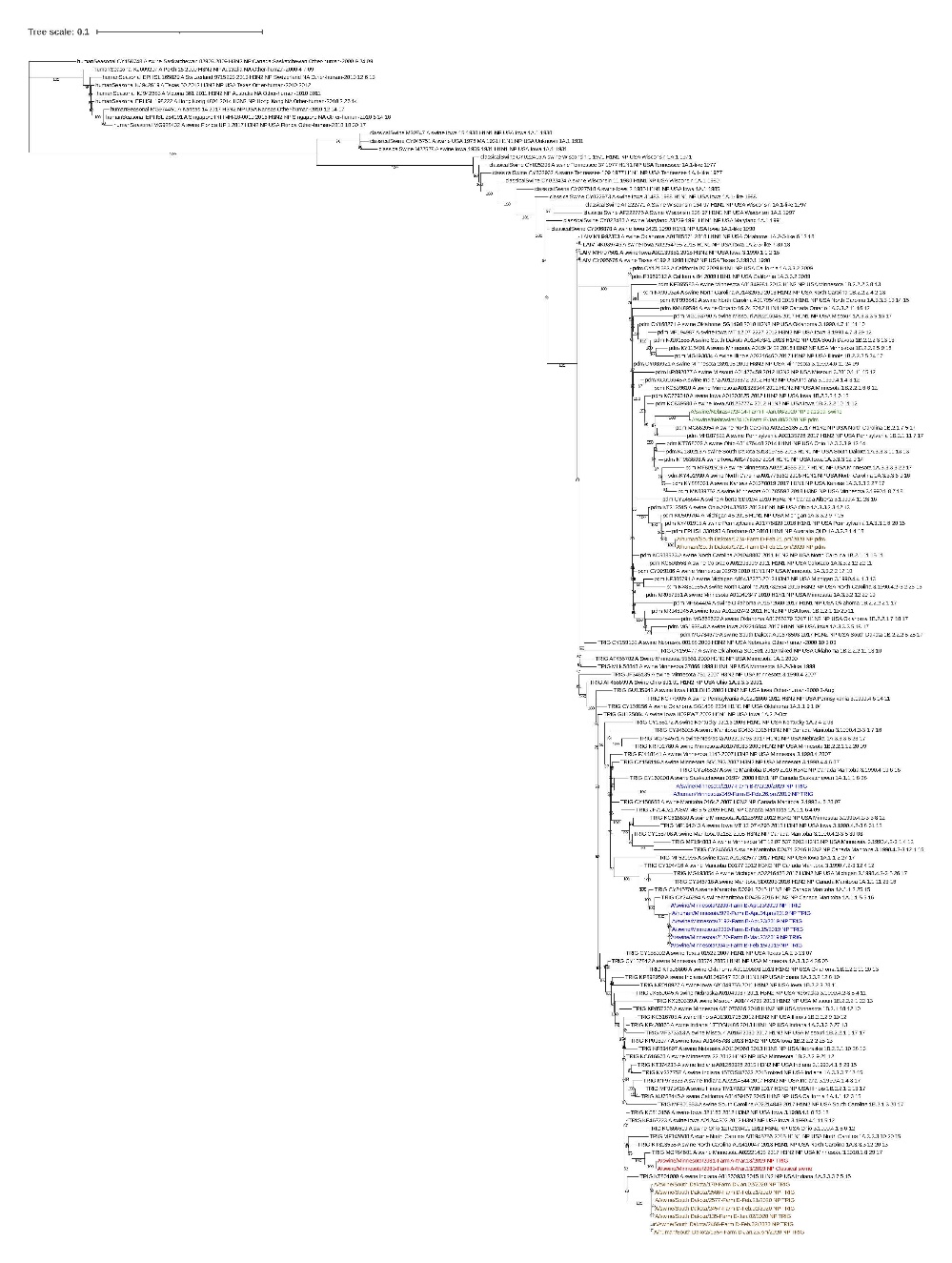


Appendix S8. Non-structural 1 gene (NS1) phylogenetic tree of study samples and references strains.


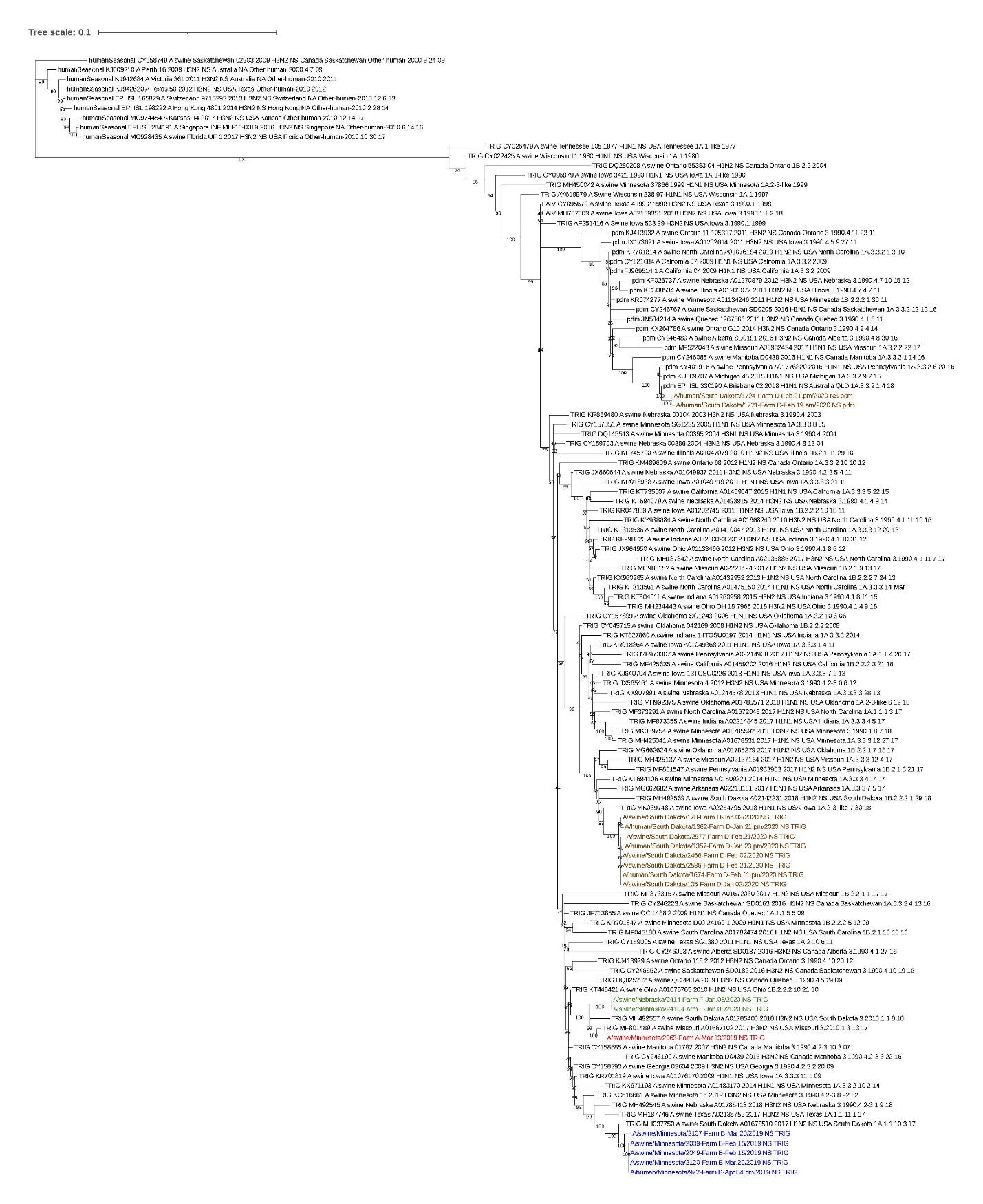


Appendix S9. Polymerase acid gene (PA) phylogenetic tree of study samples and references strains.


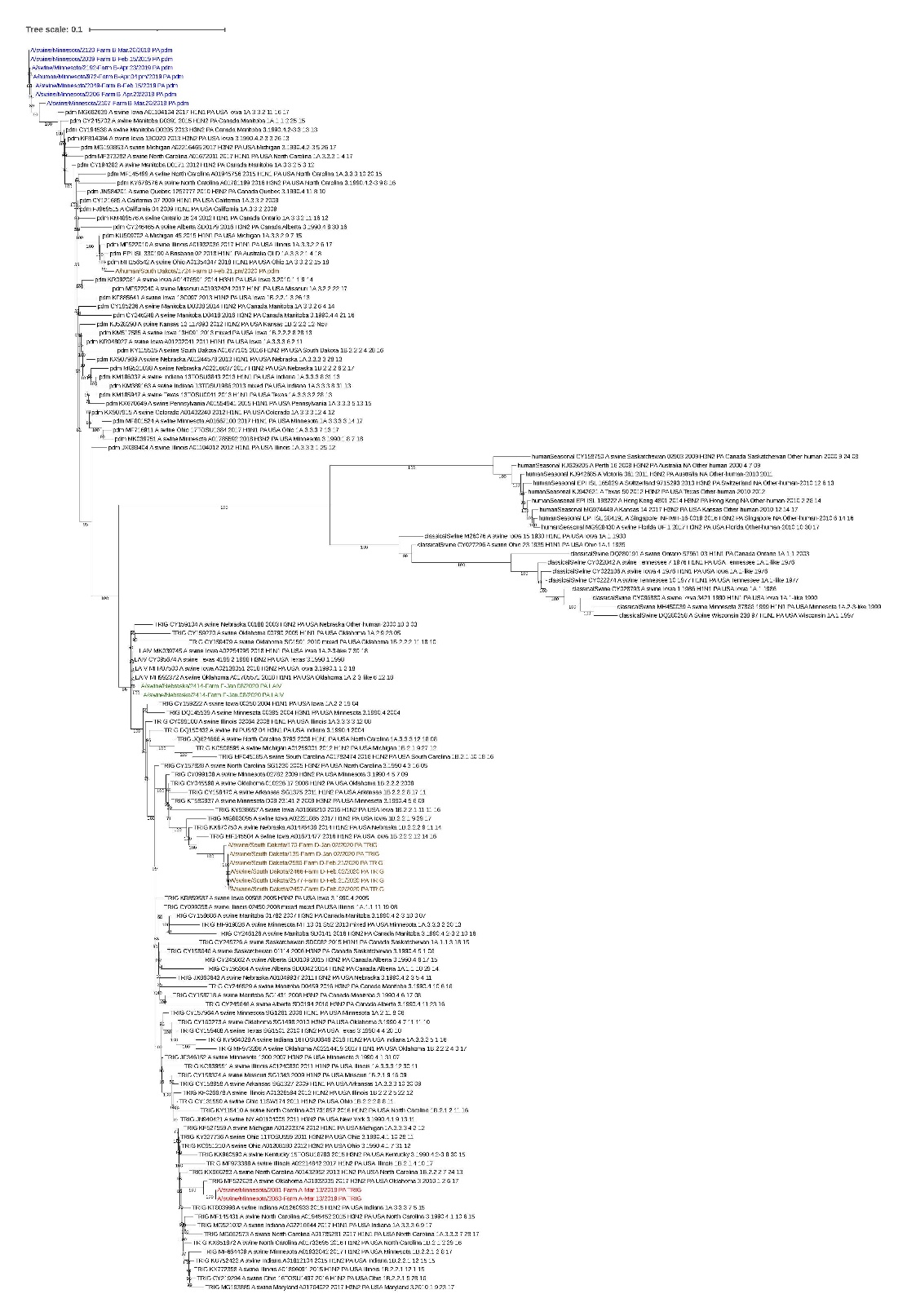


Appendix S10. Polymerase basic 1 gene (PB1) phylogenetic tree of study samples and references strains.


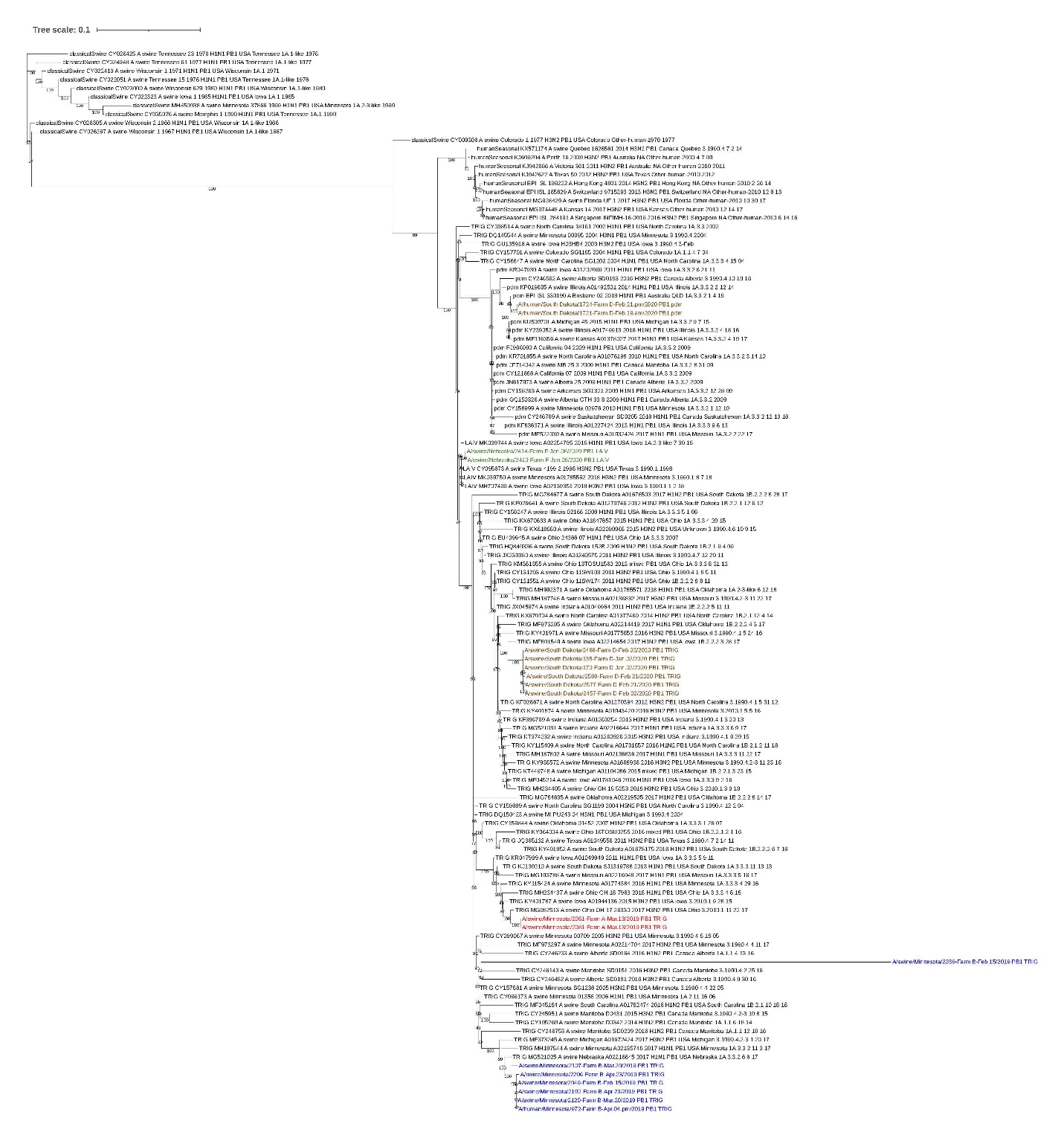


Appendix S11. Polymerase basic 2 gene (PB2) phylogenetic tree of study samples and references strains.


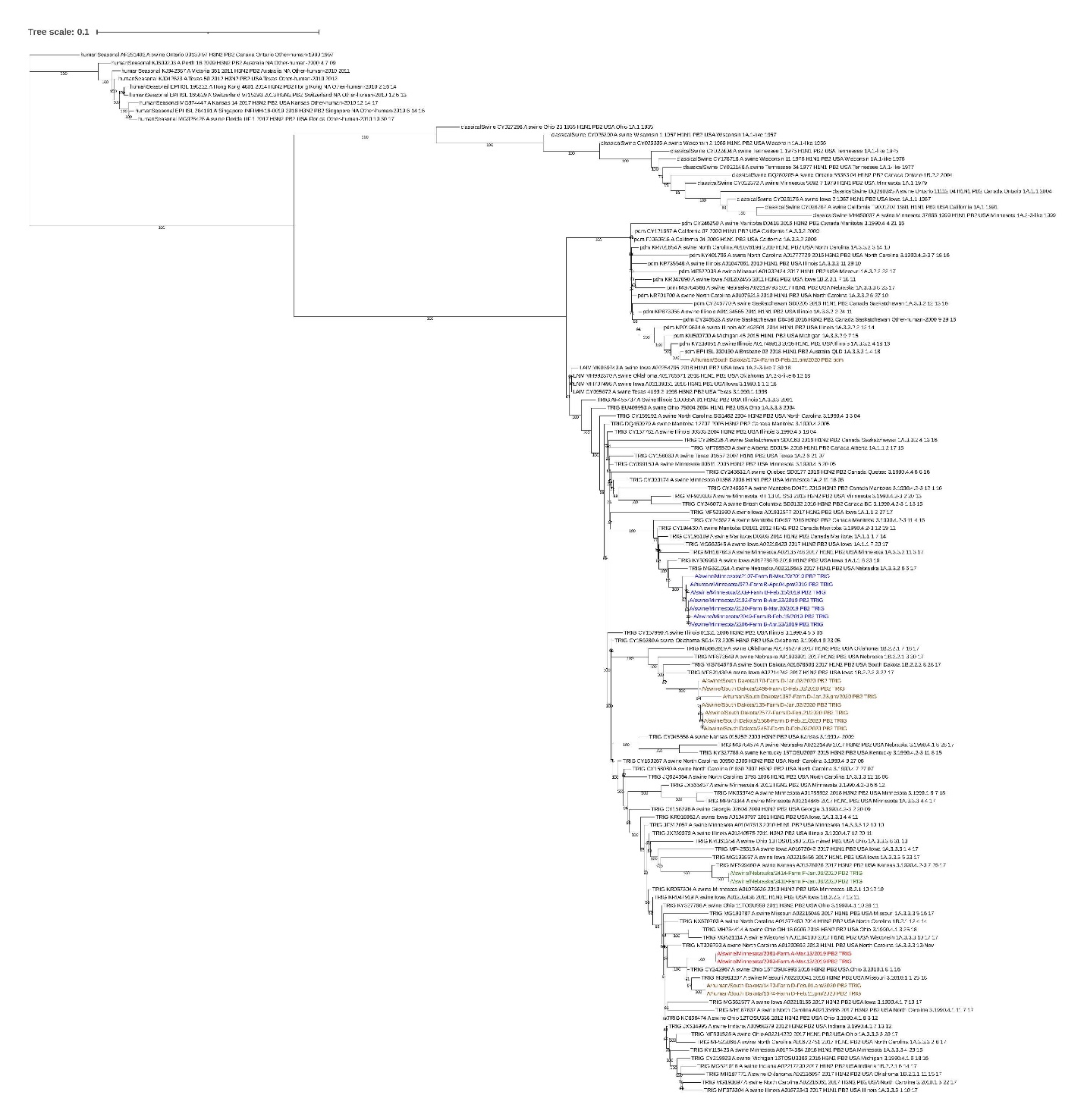


Appendix S12. List of viruses from GenBank with the highest sequence homology to each gene obtained from farmworker number 49 from whom a human seasonal H1N1 virus was detected.

| **Gene** | **Positions** | **Virus with highest percentage of nucleotide identity** | **Accession numbers** | **Homology (%)**  **Sample 1721** | **Homology (%)**  **Sample 1722** | **Homology (%)**  **Sample 1724** |
| --- | --- | --- | --- | --- | --- | --- |
| PB2 | 1-2280 | A/Human/Wyoming/04/2020(H1N1) | [[MT331565.1](https://www.ncbi.nlm.nih.gov/nucleotide/MT331565.1?report=genbank&log$=nucltop&blast_rank=1&RID=D4FWXMUG016)](https://www.ncbi.nlm.nih.gov/nucleotide/CY095672.1?report=genbank&log$=nucltop&blast_rank=1&RID=REJ0Z47Z016) | - | - | 99.94 |
| PB1 | 1-2277 | A/Human/Baltimore/R0731/2020(H1N1) | [MW843870.1](https://www.ncbi.nlm.nih.gov/nucleotide/MW843870.1?report=genbank&log$=nucltop&blast_rank=1&RID=D4G3S2NK013) | 100 | - | 100 |
| PA | 1-2151 | A/Human/Hawaii/27/2020(H1N1) | [MT622898.1](https://www.ncbi.nlm.nih.gov/nucleotide/MT622898.1?report=genbank&log$=nucltop&blast_rank=1&RID=D4G93Z7N016) | - | - | 100 |
| HA | 1-1701 | A/Human/Rochester/U135/2020(H1N1) | [MW904771.1](https://www.ncbi.nlm.nih.gov/nucleotide/MW904771.1?report=genbank&log$=nucltop&blast_rank=1&RID=D4E579GG013) | 99.74 | - | 99.77 |
| NP | 1-1497 | A/Human/New York City/PV08030/2020(H1N1) | [MW855318.1](https://www.ncbi.nlm.nih.gov/nucleotide/MW855318.1?report=genbank&log$=nucltop&blast_rank=1&RID=D4GC1MM9016) | 100 | - | 100 |
| NA | 1-1410 | A/Human/New York City/PV08030/2020(H1N1) | [MW855317.1](https://www.ncbi.nlm.nih.gov/nucleotide/MW855317.1?report=genbank&log$=nucltop&blast_rank=1&RID=D4FKYATR013) | 99.86 | 99.79 | 99.93 |
| M | 1-982 | A/Human/Baltimore/R0718/2020(H1N1 | [MW841330.1](https://www.ncbi.nlm.nih.gov/nucleotide/MW841330.1?report=genbank&log$=nucltop&blast_rank=1&RID=D4GGSNWJ013) | 100 | 99.45 | 99.90 |
| NS | 1-838 | A/Human/New York City/PV08767/2020(H1N1) | [MW855459.1](https://www.ncbi.nlm.nih.gov/nucleotide/MW855459.1?report=genbank&log$=nucltop&blast_rank=1&RID=D4GR0ZXZ013) | 99.78 | - | 99.89 |

Appendix S13: Odds ratios (OR) of farmworkers testing influenza A virus (IAV) positive using a univariate logistic regression model. Time of collection, influenza-like illness (ILI) symptoms, farm area, vaccination status and farm IAV status were used as predictor variables.

| Variable | Category | Number of positive samples/Total samples (%) | Β^+^ | SE^#^ | OR (95% CI)^ | P value |
| --- | --- | --- | --- | --- | --- | --- |
| Time of collection | Before work | 20/898 (2.2) | Reference |  | - | - |
|  | After work | 38/887 (4.3) | 0.69 | 0.28 | 1.99 (1.16-3.51) | 0.01 |
| ILI-Symptoms | No | 48/1,249 (3.8) | Reference |  | - | - |
|  | Yes | 10/536 (1.9) | -0.74 | 0.35 | 0.48 (0.23 – 0.91) | 0.04 |
| Farm area | Breeding | 14/360 (3.9) | Reference |  | - | - |
|  | Farrowing | 26/717 (3.6) | -0.03 | 0.34 | 0.97 (0.51 – 1.92) | 0.92 |
|  | Mix | 18/708 (2.5) | -0.39 | 0.36 | 0.67 (0.33 – 1.39) | 0.27 |
| IAV vaccinated | No | 36/1,071 (3.4) | Reference |  | - | - |
|  | Yes | 22/714 (3.1) | -0.1 | 0.27 | 0.9 (0.52 – 1.53) | 0.72 |
| Farm IAV status | Negative | 2/132 (1.5) | Reference |  | - | - |
|  | Positive | 56/1,653 (3.4) | 0.92 | 0.72 | 2.52 (0.78 – 15.48) | 0.2 |
| ^+^Model estimate  ^#^Standard error  ^Odds ratio (95% confidence interval) | | | | | | |
